# Long-Term Impact of COVID-19 on Heart Rate Variability: A Systematic Review of Observational Studies

**DOI:** 10.3390/healthcare11081095

**Published:** 2023-04-11

**Authors:** Hyo-Weon Suh, Chan-Young Kwon, Boram Lee

**Affiliations:** 1 Health Policy Research Team, Division of Healthcare Research, National Evidence-Based Healthcare Collaborating Agency, 400 Neungdong-ro, Gwangjin-gu, Seoul 04933, Republic of Korea; hyoweonsuh@neca.re.kr; 2 Department of Oriental Neuropsychiatry, College of Korean Medicine, Dong-Eui University, Busan 47227, Republic of Korea; 3 KM Science Research Division, Korea Institute of Oriental Medicine, 1672 Yuseong-daero, Yuseong-gu, Daejeon 34054, Republic of Korea; qhfka9357@kiom.re.kr

**Keywords:** COVID-19, SARS-CoV-2, post-acute COVID-19 syndrome, HRV, long COVID, SDNN

## Abstract

Coronavirus disease 2019 (COVID-19) sequelae (or long COVID) has become a clinically significant concern. Several studies have reported the relationship between heart rate variability (HRV) parameters and COVID-19. This review investigates the long-term association between COVID-19 and HRV parameters. Four electronic databases were searched up to 29 July 2022. We included observational studies comparing HRV parameters (measurement durations: 1 min or more) in participants with and without a history of COVID-19. We used assessment tools developed by the National Heart, Lung, and Blood Institute group to evaluate the methodological quality of included studies. Eleven cross-sectional studies compared HRV parameters in individuals who recovered from acute COVID-19 infection to controls (n = 2197). Most studies reported standard deviation of normal-to-normal intervals (SDNN) and root mean square of the successive differences. The methodological quality of the included studies was not optimal. The included studies generally found decreased SDNN and parasympathetic activity in post-COVID-19 individuals. Compared to controls, decreases in SDNN were observed in individuals who recovered from COVID-19 or had long COVID. Most of the included studies emphasized parasympathetic inhibition in post-COVID-19 conditions. Due to the methodological limitations of measuring HRV parameters, the findings should be further validated by robust prospective longitudinal studies.

## 1. Introduction

Coronavirus disease 2019 (COVID-19) caused by infection with severe acute respiratory syndrome coronavirus 2 (SARS-CoV-2) has resulted in a global pandemic after the first outbreak on December 2019. According to a cohort study, the major symptoms of COVID-19 are fever, cough, rhinorrhea with/without nasal congestion, myalgia, headache, and fatigue; loss of taste or smell before the COVID-19 test and emotional disturbance is commonly reported by patients [1]. Some individuals persistently suffer from various symptoms, including dyspnea, fatigue/malaise, myalgia, headache, chest pain, cognitive impairment (i.e., brain fog), depression, and anxiety, after recovery from SARS-CoV-2 infection [2,3,4]. Therefore, a set of persistent or new symptoms after COVID-19 is called “long COVID” or “post-acute COVID-19 syndrome.” According to a WHO-led Delphi consensus, long COVID refers to a complex symptom cluster developing 3 months from the onset of COVID-19 that last more than 2 months and cannot be explained by an alternative diagnosis [5]. Common symptoms include fatigue, shortness of breath, and cognitive dysfunction; these symptoms fluctuate or relapse over time [5]. According to a meta-analysis of 50 studies, the prevalence of the post-COVID-19 condition or long COVID (symptomatic at 28+ days from the infection) reached 43% [6]. Thus, the long-term impact of COVID-19 is a cause for concern.

There is increasing attention on the cardiovascular consequences of COVID-19 after reports of severe cardiovascular manifestations in some COVID-19 patients [7,8]. Some studies found cardiovascular implications associated with prognosis and mortality in COVID-19 [9]. Similar to pulmonary manifestations, COVID-19 may cause acute and chronic damage to the cardiovascular system [10]. According to a US-based cohort study, COVID-19 survivors had significantly higher risks of the following cardiovascular diseases compared to contemporary controls: stroke (hazard ratio (HR) = 1.52 [95% confidence intervals (CIs) 1.43–1.62]); acute coronary disease (HR = 1.72 [1.56–1.90]); myocardial infarction (HR = 1.63 [1.51–1.75]); myocarditis (HR = 5.38 [3.80–7.59]); pericarditis (HR =1.85 [1.61–2.13]); and heart failure (HR = 1.72 [1.65–1.80]) [11]. Palpitations/arrhythmia and dysautonomia are frequently reported cardiovascular symptoms of long COVID [12,13,14]. For example, a case series (n = 20) showed that 75% of COVID-19 survivors had postural orthostatic tachycardia syndrome (POTS) [15], and 85% of patients had residual autonomic symptoms until 6 to 8 months after COVID-19. According to the large-scale online survey mentioned above, 30.65% of the responders experience POTS-like symptoms after COVID-19 [4].

Heart rate variability (HRV) is considered a non-invasive, objective, and validated measurement method for evaluating cardiovascular health and autonomic nervous system (ANS) function [16]. Accordingly, HRV has also been considered as an index for some clinical conditions related to ANS dysfunction, including POTS [17], the posttraumatic stress disorder [18], and the somatic symptom disorders [19]. Additionally, several studies have reported that HRV was associated with health-related outcomes in COVID-19 [20,21,22,23]. In this regard, some studies suggest that monitoring HRV can improve the management of patients with COVID-19 and their clinical outcomes [24,25]. Importantly, it has been hypothesized that the symptoms and signs of long COVID overlap with those of ANS dysfunction, such as POTS, and that this pattern may be explained by the autonomic instability [26]. Although other underlying mechanisms of long COVID include immune dysregulation, gut microbiota imbalance, and autoimmunity, ANS dysfunction is still considered a promising therapeutic target for this clinical condition [27]. Although there has been a recent systematic review investigating changes in HRV parameters in individuals currently positive for SARS-CoV-2 [28], there has been no attempt to comprehensively investigate the relationship between HRV parameters and the long-term effects of SARS-CoV-2 infection, including long COVID.

Therefore, this systematic review of observational studies aimed to investigate the long-term association between COVID-19 and HRV parameters.

## 2. Materials and Methods

This review was conducted according to the Preferred Reporting Items for Systematic Reviews and Meta-Analyses (PRISMA) statement [29] (Supplementary File S1). This research was registered in Open Science Framework (OSF) registries (https://osf.io/xpzw4 accessed on 24 January 2023).

### 2.1. Search Strategy

We conducted a systematic search of the following electronic bibliographic databases: four electronic databases (MEDLINE [via PubMed]; EMBASE [via Elsevier]; PsycARTICLES [via ProQuest]; and Cumulative Index to Nursing and Allied Health Literature [via EBSCO] on 29 July 2022. The search strategy is presented in Supplementary File S2. Moreover, a manual search on Google Scholar was conducted for the gray and potentially missing literature.

### 2.2. Eligibility Criteria and Study Selection

The eligibility criteria of this systematic review can be summarized in the following PICOS format: (1) **Population**: Individuals who recovered from COVID-19 or individuals with long COVID were included as the population of interest in this review. The recovery usually means the negative conversion of SARS-CoV-2 infection. Moreover, long COVID was defined as clinical symptoms developing at usually 3 months from the onset of COVID-19 and existing for more than 2 months [5]. Individuals who reported absent or unclear clinical symptoms after COVID-19 recovery were not excluded, but they were analyzed separately from those with long COVID. There was no restriction on the participant’s clinical condition, language, sex, age, or ethnicity; (2) **Intervention**: Not applicable; (3) **Comparator**: Healthy (or uninfected) individuals were included as control groups; (4) **Outcome**: The outcomes of interest in this review were HRV parameters. The parameters are classified into time-domain measures and frequency-domain measures. The HRV time-domain measures include the standard deviation of normal-to-normal RR intervals (SDNN), root mean square of the successive differences (RMSSD), the standard deviation of the average NN intervals for each 5 min segment of a 24 h HRV recording (SDANN), SDNN index, and percentage of successive RR intervals that differ by more than 50 ms (pMM50) [30]. On the other hand, HRV frequency-domain measures include the low-frequency band (LF), the high-frequency band (HF), and LF/HF ratio [30]. For measurement of HRV, 24-h Holter monitoring is considered the gold standard, but short-term recordings are also acceptable [16]. Nevertheless, given that recordings longer than 1 min are required to assess some HRV frequency-domain measures, including HF [16], studies with HRV measurement durations of less than 1 min were excluded. In addition, studies in which the HRV measurement durations were unclearly described were also excluded; (5) **Study design**: Observational studies, including observational cohort studies, cross-sectional studies, and case-control studies, were included in this review. However, case reports and case series were excluded. Furthermore, animal studies, reviews, and intervention studies were excluded from this review.

Two independent researchers (C.-Y.K., B.L.) performed the study selection. Disagreements between researchers were resolved by discussion. When two or more documents reported the same study (e.g., conference abstract and journal article) or if the same data was duplicated, a more detailed reference was selected, and if different data were published separately, multiple references were included. Bibliographic information was managed using the software EndNote 20 (Clarivate Analytics, Philadelphia, PA, USA).

### 2.3. Data Collection and Data Items

Two independent researchers (H.-W.S., C.-Y.K.) performed the data extraction processes. Discrepancies between the extracted data were resolved by agreement between the researchers. We extracted study characteristics, participant characteristics, data for assessing quality assessment, and HRV parameters among the included studies. The extracted data from eligible studies were entered into a Microsoft Excel file (Microsoft, Redmond, WA, USA).

### 2.4. Study Risk of Bias Assessment

Methodological quality assessments of the studies included in this review were assessed using tools developed by the National Heart, Lung, and Blood Institute group according to the study design: Quality Assessment Tool for Observational Cohort and Cross-Sectional Studies or Quality Assessment Tool of Case-Control Studies [31]. Two independent researchers (H.-W.S., C.-Y.K.) performed the quality assessment process. Disagreements between the researchers were resolved by discussion.

### 2.5. Data Analysis

Considering the heterogeneity of the type of population, time from the COVID-19 outbreak, and potential comorbid diseases, quantitative synthesis was not planned. Instead, changes in HRV parameters in individuals after COVID-19 recovery were qualitatively investigated. The population was classified into the following three categories and analyzed: (1) individuals with long COVID (i.e., symptomatic); (2) post-COVID-19 individuals without long COVID-19 (i.e., asymptomatic); (3) individuals with the uncertain presence of long COVID (i.e., unclear). Classifying the population into these three types was expected to help understand the relationship between the long-term effects of COVID-19 and HRV parameters and the characteristics of long COVID. The differences in HRV parameters for each of these three populations are summarized in a table.

## 3. Results

### 3.1. This Study Selection

**Figure 1:**
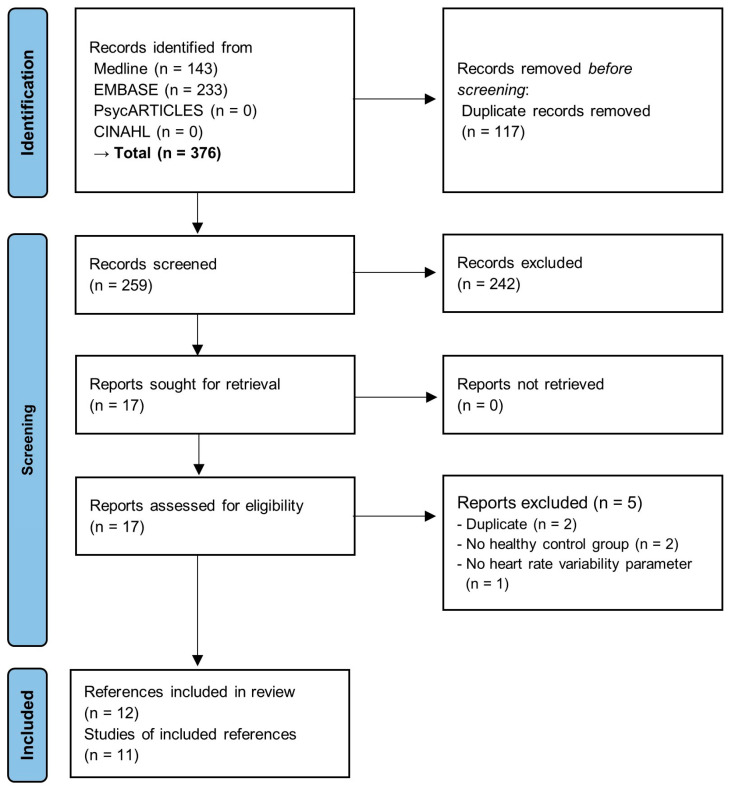
PRISMA flow diagram of the review.

A total of 376 documents were retrieved from the database. Of these, 117 duplicates were removed. The remaining 259 documents were screened for eligibility based on the titles and abstracts, and 242 irrelevant documents were excluded. The full texts of the remaining 17 documents were reviewed; of these, two were excluded due to duplication [32,33], and the other two were excluded due to the lack of a healthy (or uninfected) control group [34,35]. The last one was excluded because the study did not report HRV parameters [36]. Finally, 11 studies in 12 documents [37,38,39,40,41,42,43,44,45,46,47,48] were included in this review, of which, Mekhael et al. [45] (journal article) and Dagher et al. [48] (conference abstract) reported different outcomes for the same study. In dealing with these two documents, we described characteristics and assessed methodological quality based on Mekhael et al. [45], but we extracted HRV parameters from Dagher et al. [48]. The overall process of study selection is presented in Figure 1.

### 3.2. Characteristics of the Included Studies

All studies had a cross-sectional design. Of the 11 included studies (except for Dagher et al. [48]), 10 were published as journal articles [38,39,40,41,42,43,44,45,46,47], and 1 was presented as a conference abstract [37]. Two studies were conducted in the USA [37,45], Italy [38,47], Turkiye [40,42], and Brazil [41,44]; the remaining studies were conducted in Spain [39], China [43], and India [46]. The number of participants in each study ranged from 25 to 710. A total of 2197 participants were included; 856 (39.0%) were individuals with previous COVID-19 infection, and 1322 (60.2%) were healthy uninfected controls. The remaining 19 participants fully recovered COVID-19 controls without long COVID symptoms [39]. The mean age of the previous COVID-19 group ranged from 29.17 years to 58.6 years. Six studies evaluated the patients at 3 months or more after an acute SARS-CoV-2 infection [37,39,40,42,44,47]; Freire et al. [41] and Mekhael et al. [45] assessed the participants at 15–180 days and 171 ± 114 days after infection, respectively. The others were unclear when they evaluated HRV parameters [38,43,46]. Among these, Shah et al. [46] included recovered patients from COVID-19 within 30–45 days of enrollment. HRV parameters were analyzed based on 1 or 5-min or 24-h recordings. To assess HRV parameters, four studies used portable twelve-channel tape recorders [38,39,40,42]; three used a commercial wrist-worn sensor [41,44,45]; one used a ballistocardiography-based internet-of-medical-things system [43]; one used standard 12-lead electrocardiography machine [46]; and one used a commercial finger sensor [47]. Among the various parameters, SDNN, RMSSD, LF, and HF were most common (63.6%), followed by LF/HF ratio (54.5%) and pNN50—the proportion of the number of pairs of successive normal-to-normal RR intervals that differ by more than 50 milliseconds divided by the total number of normal-to-normal intervals—(45.5%). The overall characteristics of the included studies are summarized in Table 1. The main results of the included studies are summarized in Supplementary File S3.

### 3.3. Methodological Quality Assessment

As all studies had a cross-sectional research design, the methodological quality was assessed using Quality Assessment Tool for Observational Cohort and Cross-Sectional Studies [31]. The research question (Q1) was clearly described in all studies. The study population (Q2) was specified and defined in 10 studies, except for Adler et al. [37]. The participation rate of eligible persons (Q3) was more than 50% in six studies [38,39,42,44,45,46]. Participants were recruited from the same population (Q4) by only Acanfora et al. [38]; the other four studies used other cohort databases or recruited controls from different populations [39,40,43,45], and the remaining six studies did not describe the detailed recruitment processes of control groups [37,41,42,44,46,47]. Sample size justification (Q5), exposure assessment (Q6), and sufficient timeframe (Q7) were not conducted in all studies. Furthermore, levels of exposure (Q8) were not defined and classified in all studies. Exposure (i.e., COVID-19) measures and assessment (Q9) were only clearly described in Freire [41]; the others did not report how they confirmed COVID-19. Repeated exposure assessment (Q10) was not applicable in all studies because all studies had a cross-sectional research design. Outcome (i.e., HRV) measures (Q11) were clearly defined and valid in all studies. However, the blinding of outcome assessors (Q12) was not reported in all studies. Follow-up (Q13) was not applicable because the included studies were all cross-sectional. Statistical analyses (Q14) considering confounding variables were conducted in seven studies [39,40,41,43,45,46,47] (Table 2).

### 3.4. Long-Term Impact of COVID-19 on Heart Rate Variability

#### 3.4.1. HRV Parameters in Individuals with Long COVID (n = 3)

Acanfora et al. [38] evaluated HRV parameters in patients with long COVID symptoms and compared them to individuals without a COVID-19 history; the duration of symptoms was unclear. The SDNN (ms) was significantly lower in the patient group than in the control group (92.3 ± 24.4 vs. 127 ± 36.4, *p* = 0.0001), but the RMSSD (ms) was not significantly different between the two groups (*p* > 0.05). The HF (ms^2^) significantly decreased (4.65 ± 0.9 vs. 5.33 ± 0.9; *p* = 0.015), and the LF/HF ratio significantly increased (1.46 ± 0.27 vs. 1.23 ± 0.13, *p* = 0.001) in the patient group compared to the control group. However, the LF (ms^2^) was not significantly different between the two groups (*p* > 0.05). The researchers concluded that long COVID correlated with lower parasympathetic function (i.e., lower HF component and high LF/HF ratio). Aranyó et al. [39] compared HRV parameters among three groups: group A, long COVID patients with inappropriate sinus tachycardia (IST) persisting for more than 3 months; group B, recovered COVID-19 patients without IST; group C, uninfected controls. The LF (Hz) and HF (Hz) were significantly lower in group A than in group C (670.2 ± 380 vs. 1801.5 ± 800, *p* < 0.001; 246.0 ± 179 vs. 1048.5 ± 570, *p* < 0.001). However, the LF/HF ratio was significantly higher in group A than in group C (3.6 ± 1 vs. 2.0 ± 1, *p* = 0.040). Notably, there were no significant differences in most HRV parameters between group A and group B (all, *p* > 0.05), except for daytime pNN50 (%) and VLF (both *p* > 0.05). These results showed a decrease in most HRV parameters and ANS imbalance with decreased parasympathetic activity in long COVID patients with IST. The researchers concluded that IST was especially associated with pNN50 of individuals. Marques et al. [44] assessed HRV in long COVID patients. Of these, a subgroup with persistent long COVID symptoms for more than 3 months showed a significant increase in the SDNN (ms) compared to uninfected controls (46.83 ± 133.77 vs. 46.50 ± 29.20, *p* < 0.0001). However, the RMSSD (ms) was significantly lower in the study subgroup than in the control group (38.25 ± 35.68 vs. 54.90 ± 40.64, *p* < 0.001). However, LF (normalized unit), HF (normalized unit), and the LF/HF ratio did not differ significantly between the two groups (all, *p* > 0.05). The authors suggested that a relative increase in sympathetic activity supported by low RMSSD in their findings might be related to ANS imbalances, long COVID, and sudden death in patients with a history of COVID-19 (Table 3).

#### 3.4.2. HRV Parameters in Post-COVID-19 Individuals without Long COVID (n = 2)

Aranyó et al. [39] found that individuals without long COVID showed significantly lower daytime pNN50 (%) (10.5 ± 8 vs. 17.3 ± 10, *p* = 0.019), VLF (ms^2^) (2415.7 ± 1361 vs. 3931.1 ± 2194, *p* = 0.007), LF (ms^2^) (1093.2 ± 878 vs. 1801.5 ± 500, *p* = 0.015), and HF (ms^2^) (463.7 ± 295 vs. 1048.5 ± 570, *p* < 0.001), but not nighttime pNN50 (%) (16.6 ± 15 vs. 21.4 ± 11, *p* = 0.498) and LF/HF ratio (2.7 ± 1.3 vs. 2.0 ± 1, *p* = 0.612) compared to healthy controls. Asarcikli et al. [40] reported a significant increase in the SDNN (ms), RMSSD (ms), and HF (ms^2^). This research analyzed HRV parameters in patients with previous COVID-19 infection more than 12 weeks prior and matched healthy controls. Since the study group consisted of individuals who showed no clinical symptoms during the evaluation period, long COVID was considered non-existent. When comparing the study group and the control group, the SDNN was 155 (interquartile range [IQR], 144–177) vs. 147 (IQR, 126–166) (*p* = 0.015); the RMSSD was 41 (IQR, 27–61) vs. 31 (IQR, 22–37) (*p* = 0.002); LF was 712 (IQR, 478–946) vs. 665 (IQR, 561–1065) (*p* = 0.599); HF was 325 (IQR, 175–540) vs. 148 (IQR, 105–544) (*p* = 0.037). These results implied parasympathetic overtone and increased HRV in patients with a history of COVID-19 in the post-COVID period (Table 3).

#### 3.4.3. HRV Parameters in Individuals in Whom the Presence of Long COVID Is Uncertain (n = 7)

Adler et al. [37] investigated HRV parameters in recovered COVID-19 patients at 3 and 6 months post-discharge compared to matched controls. The results were not numerically presented, although statistical significance was tested. At 3 months post-discharge, the SDNN (ms), RMSSD (ms), and pNN50 (%) of the study group were significantly lower than those of the control group (all, *p* < 0.05). The researchers concluded that COVID-19 infection was associated with impaired parasympathetic modulation of HRV at 6 months post-discharge. Freire et al. [41] conducted a sub-analysis of the Fit-COVID study [49]. This study measured HRV parameters of previous COVID-19 patients at 15–180 days after testing positive and matched healthy controls, and further investigated the impact of body mass and physical activity on HRV. The SDNN (ms) and RMSSD (ms) were significantly lower in the study group than the control group (29.13 ± 9.37 vs. 36.17 ± 9.59, *p* = 0.0282; 24.45 (IQR, 14.40–28.55) vs. 27.40 (IQR, 23.40–33.15, *p* = 0.0452). However, LF (normalized unit), HF (normalized unit), and LF/HF ratio were not significantly different between the two groups (all, *p* > 0.05). The authors concluded that parasympathetic activity was decreased (RMSSD; *p* = 0.0452) and sympathetic activity was increased (stress index; *p* = 0.0273) in the post-COVID-19 group. Moreover, overweight/obesity and physical inactivity were associated with a negative impact on ANS activity. Kurtoğlu et al. [42] compared patients with a history of COVID-19 infection at 20.0 ± 11.4 weeks after COVID-19 confirmation and healthy controls without a history of COVID-19 and vaccination. Most HRV parameters, such as SDNN (ms), RMSSD (ms), LF (ms^2^), and HF (ms^2^), were significantly decreased in the study group. When comparing the study group and the control group, the SDNN was 122.40 ± 30.90 vs. 161.30 ± 30.80 (*p* < 0.0001); the RMSSD was 1.45 ± 0.16 vs. 1.62 ± 0.18 (*p* < 0.0001); LF was 2.71 ± 0.31 vs. 2.95 ± 0.28 (*p* < 0.0001); HF was 2.29 ± 0.33 vs. 2.62 ± 0.34 (*p* < 0.0001). LF was not significantly different between the two groups when normalized (69.60 ± 11.60 vs. 67.80 ± 13.90, *p* = 0.482). These results indicated impaired cardiac parasympathetic function in patients after COVID-19 recovery. Liu et al. [43] compared HRV parameters of discharged patients with COVID-19 and matched healthy controls. The results were not numerically presented, although statistical significance was tested. The SDNN (ms), LH (ms^2^), and HF (ms^2^) were significantly lower in the study group than in the control group (all, *p* < 0.001). Based on the findings, the authors suggested that damage to the heart or ANS caused by SARS-CoV-2 infection could be long-lasting. Mekhael et al. [45] (the HRV data extracted from Dagher et al. [48]) compared individuals with previous COVID-19 infection at 171 ± 114 days and controls who were not diagnosed with COVID-19. They found a significant decrease in mean HRV (ms) in the study group (38.9 ± 614.4 vs. 44.0 ± 619.2, *p* = 0.01). This result suggested that COVID-19 infection had a long-term effect on cardiovascular and ANS. Shah et al. [46] measured the HRV parameters of individuals who recovered from COVID-19 within 30–45 days and healthy controls. Both RMSSD (ms) and SDNN (ms) were significantly lower in the study group than in the control group (13.9 ± 11.8 vs. 19.9 ± 19.5, *p* = 0.01; 16.9 ± 12.9 vs. 22.5 ± 17.6, *p* = 0.01). Specifically, they found that among individuals post-COVID-19, the proportion of individuals with orthostatic hypotension was 13.04%, and both RMSSD and SDNN were significantly lower than in those without orthostatic hypotension (5.3 ± 3.2 vs. 15.2 ± 12.1, *p* = 0.006; 9.2 ± 6.0 vs. 18.1 ± 13.3, *p* = 0.02). The authors concluded that cardiovascular dysautonomia with lower HRV was common in COVID-19-recovered subjects, especially individuals with orthostatic hypotension. Zanoli et al. [47] assessed previous COVID-19 infections more than 12 weeks prior and matched controls. Only HRV triangular index and LF/HF ratio were investigated as HRV parameters. However, these parameters did not differ significantly between the two groups (both, *p* > 0.05) (Table 3).

## 4. Discussion

### 4.1. Principal Findings

This systematic review investigated the long-term effect of COVID-19 on HRV parameters. Eleven cross-sectional studies in 12 references [37,38,39,40,41,42,43,44,45,46,47,48] were included by systematic and comprehensive search. This review broadly divided studies under three population conditions: individuals with long COVID (i.e., symptomatic); post-COVID-19 individuals without long COVID (i.e., asymptomatic); and individuals whose presence of long COVID was uncertain (i.e., unclear).

First, changes in HRV parameters in individuals with long COVID were investigated in three studies [38,39,44]. Among them, parameters that showed consistent significant differences in at least two studies included SDNN, VLF, HF, and LF/HF ratio. Specifically, compared to the healthy control group, SDNN, VLF, and HF were significantly lower in individuals with long COVID, and LF/HF ratio was significantly higher. Regarding these differences, the studies suggest the possibility that long COVID is associated with reduced parasympathetic activity [38,39] and increased sympathetic activity [44]. Interestingly, a comparison of HRV parameters in individuals with long COVID and post-COVID-19 individuals without long COVID showed significant differences in only daytime pNN50 and VLF [39]. Based on these findings, Aranyó et al. [39] suggested that pNN50 may be associated with long COVID, especially IST. Second, changes in HRV parameters in post-COVID-19 individuals without long COVID were investigated in two studies [39,40]. Although these two studies reported four common HRV parameters, including pNN50, LF, HF, and LF/HF ratio, both studies consistently found no changes in HRV parameters. Interpretation of the results also showed differences. Aranyó et al. [39] observed reduced parasympathetic activity after COVID-19, while Asarcikli et al. [40] noted parasympathetic overtone. Furthermore, Asarcikli et al. [40] found that SDNN and RMSSD were increased compared to healthy controls in post-COVID-19 individuals without long COVID, and based on the findings, they highlighted an increase in HRV after COVID-19. However, this result contrasts with findings from most other studies included in this review [37,38,41,42,43,46]. Third, changes in HRV parameters of individuals whose presence of long COVID is uncertain were investigated in seven studies in eight documents [37,41,42,43,45,46,47,48]. Among them, parameters that showed consistent significant differences in at least four studies included SDNN and RMSSD. Specifically, compared to the healthy control group, the two parameters were significantly lower in individuals whose presence of long COVID is uncertain. Significant decreases in pNN50, SDANN, LF, and HF were also reported relatively consistently in this population. However, no case showed conflicting differences between studies in the HRV parameters. Similar discussions in most studies indicated autonomic dysfunctions, specifically impaired parasympathetic modulation of HRV [37,41,42,46] and increased sympathetic activity [41] in this population.

However, the methodological quality of the included studies was not optimal. In particular, none of the included studies justified the sample size, and since all studies were cross-sectional, there were limitations in causally investigating the changes in HRV parameters found. One of the important limitations was that among the included studies, only seven [39,40,41,43,45,46,47] were measured and appropriately adjusted for key potential confounding variables.

### 4.2. Clinical Implications

Several studies have reported an acute effect of COVID-19 on the ANS [24,50]. Specifically, a previous systematic review showed mixed results of changes in SDNN and RMSSD in patients with acute COVID-19 [51], while LF and HF were generally lower in COVID-19 patients compared to healthy controls [51]. However, this review suggests a consistent finding of reduced SDNN in the long-term impact of COVID-19 and no consistent findings in RMSSD, LF, and HF. Given that SDNN is considered a gold standard indicator for the cardiac risk [30], a possible association between the long-term effects of COVID-19 and cardiac risk may be raised. A study that followed more than 150,000 individuals with COVID-19 for one year found that the risks and burdens of cardiovascular disease are substantial in COVID-19 survivors [11]. In addition, some risk factors, including pre-existing cardiovascular comorbidities, may increase cardiovascular risk in these patients [52]. Therefore, HRV parameters, such as SDNN, which can be measured non-invasively and conveniently, are considered clinically worthy of attention for the purpose of monitoring the long-term effects of COVID-19 and the cardiovascular risk of COVID-19 survivors.

One study reported an association between the long-term effects of COVID-19 and increased SDNN and overshoot of the parasympathetic activity [40]. They suggest that the overshoot of parasympathetic activity that occurs 12 weeks after COVID-19 is reactive, and the increase in sympathetic tone during acute COVID-19 may have prevented this overshoot of parasympathetic activity [40]. However, this finding contradicts other studies [37,42] on the population during a similar period after COVID-19. Moreover, a significant increase in parasympathetic tone supported by increased SDNN and RMSSD during the acute COVID-19 period has also been reported [24]. This finding suggests that it may be premature to draw consensus conclusions about changes in ANS in the context of COVID-19. A recent review suggested that potential mechanisms of ANS dysfunction after COVID-19 may involve direct invasion of SARS-CoV-2 through neuronal or hematogenous routes, autoimmunity, persistent inflammation, hypoxia, and renin-angiotensin system imbalance [53]. In the context of the diverse mechanisms, whether ANS plays a pivotal role in post-COVID-19 conditions, including long COVID, remains to be elucidated, and the findings in this review provide only fragmented information.

The COVID-19 pandemic is considered to have triggered a huge shift in the popularization of telemedicine in the delivery of healthcare [54,55]. Moreover, the use of wearable health devices plays an important role in terms of telemedicine [54]. Importantly, HRV is one of the popular health indicators for wearable health devices [55]. Therefore, if telemedicine using wearable health devices becomes more popular in the post-COVID-19 era, establishing the clinical significance of HRV parameters from the perspective of evidence-based medicine is clinically relevant. In this context, the findings of this review could be used to promote better patient–doctor communication or to facilitate the successful implementation of wearable health devices [56].

Studies of some established post-COVID-19 conditions can strengthen the methodology of HRV research. For example, in some case reports and case series, POTS was observed in COVID-19 patients [15,57,58,59,60], with significant changes in HRV parameters [61,62]. In this review, Aranyó et al. [39] also diagnosed IST, characterized by elevated heart rate when the patients move from a supine to an upright position; this syndrome is overlapped with POTS [63]. Additionally, a cross-sectional study on 320 patients with long COVID reported that 73.6% of patients suffered from orthostatic intolerance [64]. The cardiovagal baroreflex deficits observed in POTS may be related to reduced parasympathetic withdrawal during supine and sympathetic activation in the upright position [65], which suggests that future studies evaluating HRV in post-COVID-19 conditions should investigate changes in HRV parameters according to body position changes.

### 4.3. Limitations

There were previous systematic reviews of the associations of HRV parameters with the COVID-19 vaccination [66] and with the acute COVID-19 infection [28,51]. To our knowledge, this review is the first comprehensive review of the long-term effects of COVID-19 based on HRV parameters. However, this review has several limitations. First, this review was not strictly limited to long COVID but comprehensively included the post-COVID-19 population. Since most included studies did not use the strict criteria of long COVID, our findings do not represent HRV parameters in patients with long COVID. Instead, the findings of this study should be used to understand the long-term effects of COVID-19 on HRV parameters, including long COVID. Comparisons between asymptomatic post-COVID-19 conditions and long COVID can further advance our understanding of long COVID. Second, we could not investigate causal relationships because the included studies were cross-sectional. Because the included studies only used uninfected or healthy controls as their control groups, it was not possible to analyze longitudinal changes in HRV parameters in individuals before and after SARS-CoV-2 infection. Additionally, the comparability of the controls and infected patients can also be pointed out as a limitation of this review. Although the majority of included studies adjusted for key potential confounding variables between exposed and control groups [39,40,41,43,45,46,47], there were also some studies that did not. Thus, the authors admit that the findings of this review may have been biased. Some authors of the primary studies concluded that autonomic imbalance could explain the pathology of the long COVID-19 [38,39], but this conclusion would require further longitudinal cohort studies. Third, there is heterogeneity in the devices or recording times of HRV parameter measurements in included studies. Ideally, studies on HRV parameters should use certified medical devices and follow standard guidelines to provide reliable findings [30]. Considering the characteristics of long COVID, including orthostatic intolerance (e.g., POTS), future research should focus on improving HRV measurement methods, such as measurement of HRV parameters according to body position changes. Fourth, the included studies and this review attempted to interpret ANS dysfunction in post-COVID-19 conditions based on changes in HRV parameters. However, HRV parameters, such as LF/HF ratio, do not fully represent the ANS function. Specifically, some variables, such as respiration rate, should be considered for the relationship between HRV parameters and ANS function; however, no study included the relevant variables. Hence, there are limitations in interpreting the findings of this review in relation to the ANS function. Finally, this review does not identify a potentially age-dependent impact of post-COVID-19 conditions on HRV parameters. Depending on the age of individuals included in the studies, it could have been classified as children and adolescents (i.e., under 18), adults (i.e., 18 to 65), and the elderly (i.e., over 65), but among the included studies, there were no studies targeting children, adolescents, or the elderly. As the mean age of individuals in the included studies ranged from 26 to 59 years, age stratification was not possible to allow for analysis by age group. However, the long-term effects of age-dependent SARS-CoV-2 infection deserve further exploration. ANS function was found to decline in old age compared to young adults or middle age [67]. In addition, it is known that SDNN and SDANN show a linear decrease with age, while RMSSD and pNN50 show a reversal increase at the age of 60 or older [68]. Importantly, an analysis of 205 patients referred to the post-COVID-19 service found that the mean age of individuals with dysautonomia was statistically significantly older compared to individuals without dysautonomia (*p* < 0.001) [69]. Although there is a systematic review investigating long COVID in children and adolescents [70], the difference in long COVID by age group and the potential relevance of HRV or ANS function should be further investigated in the future.

## 5. Conclusions

Compared to uninfected or healthy controls, decreases in SDNN were observed in individuals who recovered from COVID-19 or had long COVID. On the other hand, consistent differences between the groups were not observed in RMSSD, LF, and HF. Most included studies emphasized parasympathetic inhibition in post-COVID-19 conditions, including long COVID-19. However, the studies included in this review are cross-sectional and do not guarantee causality. Moreover, due to the methodological limitations of studies, including the measurement of HRV parameters, the findings should be further validated by more robustly designed prospective longitudinal studies.

## Figures and Tables

**Table 1:** Characteristics of the included studies.

Author	Country	Comparison	Population (Mean Age)	Assessment Time Point	Assessment Duration (Device)	HRV Parameters
Adler 2021 [37]	USA	G1: Previous COVID-19 infection (at 3- and 6-months post-discharge) (n = 18) G2: Matched controls (n = 7)	G1: 50 ± 16 G2: 50 ± 14	3 months (12 weeks) or more	1-min HRV (unclear) responses to orthostatic stress (3-min active standing)	1. SDNN (ms); 2. RMSSD (ms); 3. pNN50 (%)
Acanfora 2022 [38]	Italy	G1: Long COVID patients (n = 30) G2: No-COVID-19 patients (n = 20)	G1: 58.6 ± 17.6 G2: 56.3 ± 14.7	unclear	24-h ECG monitoring (portable twelve-channel tape recorder)	1. SDNN (ms); 2. SDANN (ms); 3. RMSSD (ms); 4. SDNN Index (ms); 5. pNN50 (%); 6. total power (ms^2^); 7. VLF (ms^2^); 8. LF (ms^2^); 9. HF (ms^2^); 10. LF/HF ratio
Aranyó 2022 [39]	Spain	G1: Long COVID patients with IST) (n = 40) G2: Fully recovered COVID-19 patients (n = 19) G3: Uninfected controls (n = 17)	G1: 40.1 ± 10 G2: 42.2 ± 11 G3: 39.5 ± 13	3 months (12 weeks) or more	24-h ECG monitoring (AFT 1000 + B recorder)	1. Daytime SD (ms); 2. Daytime pNN50 (%); 3. Nighttime SD (ms); 4. Nighttime pNN50 (%); 5. VLF (Hz); 6. LF (Hz); 7. HF (Hz); 8. LF/HF ratio
Asarcikli 2022 [40]	Turkiye	G1: Previous COVID-19 infection (>12 weeks) and no current clinical symptoms (n = 60) G2: Matched healthy controls (n = 33)	G1: 39 (range 31–49) G2: 30 (range 26–42)	3 months (12 weeks) or more	24-h ECG monitoring (DMS300-4A Holter ECG recorder)	1. SDNN (ms); 2. SDANN (ms); 3. RMSSD (ms); 4. SDNN Index (ms); 5. pNN50 (%); 6. total power (ms^2^); 7. LF (ms^2^); 8. HF (ms^2^); 9. LF/HF ratio; 10. SDNN > 60 ms; 11. RMSSD > 40 ms
Freire 2022 [41]	Brazil	G1: Previous COVID-19 infection (at 15–180 days) (n = 20) G2: Matched healthy controls (n = 18)	G1: 29.17 ± 6.32 G2: 26.22 ± 5.22	15–180 days	5-min HRV (Polar RS800CX)	1. SDNN (ms); 2. RMSSD (ms); 3. pNN50 (%); 4. LF (nu); 5. HF (nu); 6. LF/HF ratio; 7. Triangular index; 8. TINN (ms)
Kurtoğlu 2022 [42]	Turkiye	G1: Patients with a confirmed history of COVID-19 (at 20.0 ± 11.4 weeks) (n = 50) G2: Healthy controls without a history of COVID-19 and vaccination (n = 50)	G1: 40.82 ± 10.31 G2: 38.24 ± 12.02	3 months (12 weeks) or more	24-h ECG monitoring (iH-12Plus Holter System)	1. SDNN (ms); 2. SDANN (ms); 3. RMSSD (ms); 4. SDNN Index (ms); 5. pNN50 (%); 6. total power (ms^2^); 7. VLF (ms^2^); 8. LF (ms^2^); 9. HF (ms^2^); 10. LF (nu); 11. HF (nu); 12. Triangular index (HRVI)
Liu 2021 [43]	China	G1: Discharged COVID-19 patients (n = 186 → 164 analyzed) G2: Matched healthy controls (n = 186 → 166 analyzed)	Not reported	unclear	more than 10-h recording (ballistocardiography-based internet-of-medical-things system)	1. SDNN (ms); 2. SDANN (ms); 3. LF (ms^2^); 4. HF (ms^2^)
Marques 2022 [44]	Brazil	G1: Long COVID clinical group (n = 155 → 81 analyzed) G2: Uninfected controls (n = 94)	G1: 43.88 ± 10.03 G2: 40.69 ± 6.35	3 months (12 weeks) or more	5-min HRV (Polar RS800CX)	1. SDNN (ms); 2. RMSSD (ms); 3. LF (nu); 4. HF (nu); 5. LF/HF ratio; 6. SD1 (ms); 7. SD2 (ms)
Mekhael 2022 [45]; Dagher 2022 [48]	USA	G1: Previous COVID-19 infection (at 171 ± 114 days) (n = 122) G2: Controls who were not diagnosed with COVID-19 (n = 588)	G1: 41.32 ± 15.7 G2: 45.99 ± 14.0	171 ± 114 days	5-min HRV (PPG-based smartband)	1. Mean HRV day/person (ms)
Shah 2022 [46]	India	G1: Previous COVID-19 infection (recovered within 30–45 days) (n = 92) G2: Healthy volunteer controls (n = 120)	G1: 50.6 ± 12.1 G2: 51.8 ± 4.2	unclear (recovered within 30–45 days)	1-min HRV (VESTA 301i) responses to orthostatic stress (3-min active standing)	1. RMSSD (ms)
Zanoli 2022 [47]	Italy	G1: Previous COVID-19 infection (>12 weeks) (n = 92) G2: Matched controls (n = 180)	G1: 55 ± 12 G2: 55 ± 13	3 months (12 weeks) or more	5-min HRV (Finometer Midi device)	1. LF/HF ratio; 2. Triangular index

**Abbreviations**. COVID-19, Coronavirus disease 2019; ECG, electrocardiogram; G, group; HF, high-frequency band; HRV, heart rate variability; IST, inappropriate sinus tachycardia; LF, low-frequency band; nu, normalized unit; pNN50, proportion of the number of pairs of successive normal-to-normal RR intervals that differ by more than 50 milliseconds divided by the total number of normal-to-normal RR intervals; RMSSD, root mean square of the successive differences; SD, standard deviation of the interbeat interval; SDANN, standard deviation of the averages of normal-to-normal RR intervals; SDNN, standard deviation of normal-to-normal RR intervals; SDNN index, mean of the standard deviations of all normal-to-normal RR intervals for all 5 min segments of the entire recording; TINN, triangular interpolation of normal-to-normal RR intervals; VLF, very low-frequency band.

**Table 2:** Methodological quality of the included studies.

Author	Q1	Q2	Q3	Q4	Q5	Q6	Q7	Q8	Q9	Q10	Q11	Q12	Q13	Q14
Adler 2021 [37]	Y	N	NR	CD	N	N	N	NA	NR	NA	Y	NR	NA	NR
Acanfora 2022 [38]	Y	Y	Y	Y	N	N	N	NA	NR	NA	Y	NR	NA	N
Aranyó 2022 [39]	Y	Y	Y	N	N	N	N	NA	NR	NA	Y	NR	NA	Y
Asarcikli 2022 [40]	Y	Y	NA	N	N	N	N	NA	NR	NA	Y	NR	NA	Y
Freire 2022 [41]	Y	Y	N	CD	N	N	N	NA	Y	NA	Y	NR	NA	Y
Kurtoğlu 2022 [42]	Y	Y	Y	CD	N	N	N	NA	NR	NA	Y	NR	NA	NR
Liu 2021 [43]	Y	Y	NR	N	N	N	N	NA	NR	NA	Y	NR	NA	Y
Marques 2022 [44]	Y	Y	Y	CD	N	N	N	NA	NR	NA	Y	NR	NA	NR
Mekhael 2022 [45]; Dagher 2022 [48]	Y	Y	Y	N	N	N	N	NA	NR	NA	Y	NR	NA	Y
Shah 2022 [46]	Y	Y	Y	CD	N	N	N	NA	NR	NA	Y	NR	NA	Y
Zanoli 2022 [47]	Y	Y	NA	CD	N	N	N	NA	NR	NA	Y	NR	NA	Y

**Abbreviations**. CD, cannot determine; N, no; NA, not applicable; NR, not reported; Y, yes. **Note**: Q1. Was the research question or objective in this paper clearly stated?; Q2. Was the study population clearly specified and defined?; Q3. Was the participation rate of eligible persons at least 50%?; Q4. Were all the subjects selected or recruited from the same or similar populations (including the same time period)? Were inclusion and exclusion criteria for being in the study prespecified and applied uniformly to all participants?; Q5. Was a sample size justification, power description, or variance and effect estimates provided?; Q6. For the analyses in this paper, was the exposure(s) of interest measured prior to the outcome(s) being measured?; Q7. Was the timeframe sufficient so that one could reasonably expect to see an association between exposure and outcome if it existed?; Q8. For exposures that can vary in amount or level, did the study examine different levels of exposure as related to the outcome (e.g., categories of exposure or exposure measured as continuous variable)?; Q9. Were the exposure measures (independent variables) clearly defined, valid, reliable, and implemented consistently across all study participants?; Q10. Was the exposure(s) assessed more than once over time?; Q11. Were the outcome measures (dependent variables) clearly defined, valid, reliable, and implemented consistently across all study participants?; Q12. Were the outcome assessors blinded to the exposure status of participants?; Q13. Was loss to follow-up after baseline 20% or less?; Q14. Were key potential confounding variables measured and adjusted statistically for their impact on the relationship between exposure(s) and outcome(s)?

**Table 3:** Long-term impact of COVID-19 on heart rate variability.

Study	Time Domain							Frequency Domain						
	SDNN (ms)	RMSSD (ms)	pNN50 (%)	SDANN (ms)	SDNN Index (ms)	Triangular Index	Mean HRV (ms)	VLF (ms^2^)	LF (ms^2^)	LF (nu)	HF (ms^2^)	HF (nu)	LF/HF Ratio	Total Power (ms^2^)
Individuals with long COVID vs. healthy controls (vs. post-COVID-19 individuals without long COVID)														
Acanfora 2022 [38] *	−	NS	NS	−	−			−	NS		−		+	−
Aranyó 2022 [39] *			D: −, N: − (D: −, N: NS)					− (−)	− (NS)		− (NS)		+ (NS)	
Marques 2022 [44]	−	+								NS		NS	NS	
Post-COVID-19 individuals without long COVID vs. healthy controls														
Asarcikli 2022 [40] *	+	+	+	+	+				NS		+		−	NS
Aranyó 2022 [39] *			D: −, N: NS					−	−		−		NS	
Individuals whose presence of long COVID is uncertain vs. healthy controls														
Adler 2021 [37]	−	−	−											
Freire 2022 [41]	−	−	NS			NS			NS		NS		NS	
Kurtoğlu 2022 [42] *	−	−	−	−	−	−		−	−	NS	−	−		
Liu 2021 [43]	−			−					−		−			
Mekhael 2022 [45]; Dagher 2022 [48]							−							
Shah 2022 [46]		−												
Zanoli 2022 [47]						NS							NS	

**Abbreviations**. COVID-19, coronavirus disease 2019; D, daytime; ECG, electrocardiogram; HF, high-frequency band; HRV, heart rate variability; LF, low-frequency band; N, nighttime; NS, not significant; nu, normalized unit; pNN50, proportion of the number of pairs of successive normal-to-normal RR intervals that differ by more than 50 milliseconds divided by the total number of normal-to-normal RR intervals; RMSSD, root mean square of the successive differences; SDANN, standard deviation of the averages of normal-to-normal RR intervals; SDNN, standard deviation of normal-to-normal RR intervals; VLF, very low-frequency band. **Note**: ‘+’ means a statistically significant increase compared to the control, while ‘−’ means a statistically significant decrease compared to the control. ‘*’ indicates a study in which HRV was recorded using 24-h ECG monitoring.

## Data Availability

The data used to support the findings of this study are included within the article.

## Supplementary Materials

The following supporting information can be downloaded at: https://www.mdpi.com/article/10.3390/healthcare11081095/s1, Supplementary File S1: PRISMA 2020 Checklist; Supplementary File S2: Search strategies used in each database; Supplementary File S3: Main findings of included studies.
