# Supplementary material for: Long-Term Impact of COVID-19 on Heart Rate Variability: A Systematic Review of Observational Studies
Source: Healthcare (Basel). 2023 Apr 11;11(8):1095. doi: 10.3390/healthcare11081095 (PMC10137929; doi:10.3390/healthcare11081095)
Supplement: Supplementary file 1 [file healthcare-11-01095-s001.zip › healthcare-2327082-supplementary/Supplementary S3.pdf]

Supplementary S3. Main findings of included studies.

Author	Study type	Comparison	Population (mean age)	Assessment time point	Assessment duration (device)	HRV parameters	Findings G1 vs. G2 (vs. G3)
Adler 2021	cross-sectional study	G1: Previous COVID-19 infection (at 3- and 6-months post-discharge) (n = 18)<br>G2: Matched controls (n = 7)	G1: 50 ± 16<br>G2: 50 ± 14	3 months (12 weeks) or more	1-min HRV (unclear) responses to orthostatic stress (3-min active standing)	1. SDNN (ms); 2. RMSSD (ms); 3. pNN50 (%)	Law data was not reported.<br>long COVID vs. controls ($p < 0.05$)<br>1. SDNN (ms): <<br>2. RMSSD (ms): <<br>3. pNN50 (%): <
Acanfora 2022	cross-sectional study	G1: Long COVID patients (n = 30)<br>G2: No-COVID-19 patients (n = 20)	G1: 58.6 ± 17.6<br>G2: 56.3 ± 14.7	unclear	24-h ECG monitoring (portable twelve-channel tape recorder)	1. SDNN (ms); 2. SDANN (ms); 3. RMSSD (ms); 4. SDNN Index (ms); 5. pNN50 (%); 6. total power (ms^2); 7. VLF (ms^2); 8. LF (ms^2); 9. HF (ms^2); 10. LF/HF ratio	1. 92.3 ± 24.4 vs. 127 ± 36.4 (p = 0.0001)<br>2. 79 ± 21.9 vs. 109.9 ± 36.8 (p = 0.001)<br>3. 24.5 ± 12.3 vs. 33.9 ± 20.9 (p > 0.05)<br>4. 41.9 ± 15.3 vs. 57.6 ± 14.5 (p = 0.001)<br>5. 5.7 ± 7.8 vs. 10.8 ± 11.2 (p > 0.05)<br>6. 7.46 ± 0.5 vs. 8.08 ± 0.6 (p < 0.0001)<br>7. 6.84 ± 0.8 vs. 7.66 ± 0.6 (p < 0.0001)<br>8. 6.55 ± 0.42 vs. 6.44 ± 0.74 (p > 0.05)<br>9. 4.65 ± 0.9 vs. 5.33 ± 0.9 (p = 0.015)<br>10. 1.46 ± 0.27 vs. 1.23 ± 0.13 (p = 0.001)
Aranyó 2022	cross-sectional study	G1: Long COVID patients with IST) (n = 40)<br>G2: Fully recovered COVID-19 patients (n = 19)<br>G3: Uninfected controls (n = 17)	G1: 40.1 ± 10<br>G2: 42.2 ± 11<br>G3: 39.5 ± 13	3 months (12 weeks) or more	24-h ECG monitoring (AFT 1000 + B recorder)	1. Daytime SD (ms); 2. Daytime pNN50 (%); 3. Nighttime SD (ms); 4. Nighttime pNN50 (%); 5. VLF (Hz); 6. LF (Hz); 7. HF (Hz); 8. LF/HF ratio	(p-value: IST vs. recovered, IST vs. uninfected)<br>1. 95.0 ± 25 vs. 121.5 ± 34 vs. 138.1 ± 25 (p = 0.011, p < 0.001)<br>2. 3.2 ± 3 vs. 10.5 ± 8 vs. 17.3 ± 10 (p = 0.001, p < 0.001)<br>3. 101.3 ± 28 vs. 144.5 ± 42 vs. 145.4 ± 39 (p < 0.001, p = 0.003)<br>4. 8.4 ± 8 vs. 16.6 ± 15 vs. 21.4 ± 11 (p = 0.051, p = 0.004)<br>5. 1463.1 ± 538 vs. 2415.7 ± 1361 vs. 3931.1 ± 2194 (p = 0.044, p < 0.001)<br>6. 670.2 ± 380 vs. 1093.2 ± 878 vs. 1801.5 ± 800 (p = 0.092, p < 0.001)<br>7. 246.0 ± 179 vs. 463.7 ± 295 vs. 1048.5 ± 570 (p = 0.060, p < 0.001)<br>8. 3.6 ± 1 vs. 2.7 ± 1.3 vs. 2.0 ± 1 (p = 0.259, p = 0.040)
Asarcikli 2022	cross-sectional study	G1: Previous COVID-19 infection (> 12 weeks) and no current clinical symptoms (n = 60)	G1: 39 (range 31–49)<br>G2: 30 (range 26–42)	3 months (12 weeks) or more	24-h ECG monitoring (DMS300-4A Holter ECG recorder)	1. SDNN (ms); 2. SDANN (ms); 3. RMSSD (ms); 4. SDNN Index (ms); 5. pNN50 (%); 6. total	1. 155 (IQR 144–177) vs. 147 (IQR 126–166) (p = 0.015)<br>2. 154 (IQR 127–166) vs. 135 (IQR 114–154) (p = 0.041)<br>3. 41 (IQR 27–61) vs. 31 (IQR 22–37) (p = 0.002)<br>4. 64 (IQR 54–97) vs. 53 (IQR 47–64) (p = 0.003)<br>5. 14 (IQR 11–18) vs. 9 (IQR 3–16) (p = 0.032)

		G2: Matched healthy controls (n = 33)				power (ms^2); 7. LF (ms^2); 8. HF (ms^2); 9. LF/HF ratio; 10. SDNN > 60 ms; 11. RMSSD > 40 ms	6. 3.148 (IQR 2.348–4.408) vs. 2.854 (IQR 2.212–4.195) (p = 0.474)<br>7. 712 (IQR 478–946) vs. 665 (IQR 561–1065) (p = 0.599)<br>8. 325 (IQR 175–540) vs. 148 (IQR 105–544) (p = 0.037)<br>9. 1.99 (IQR 1.29–3.80) vs. 3.53 (IQR 1.97–5.78) (p = 0.010)<br>10. 36 (60.0%) vs. 12 (36.4%) (p = 0.028)<br>11. 31 (51.7%) vs. 7 (21.2%) (p = 0.003)
Freire 2022	cross-sectional study	G1: Previous COVID-19 infection (at 15-180 days) (n = 20)<br>G2: Matched healthy controls (n = 18)	G1: 29.17 ± 6.32<br>G2: 26.22 ± 5.22	15-180 days	5-min HRV (Polar RS800CX)	1. SDNN (ms); 2. RMSSD (ms); 3. pNN50 (%); 4. LF (nu); 5. HF (nu); 6. LF/HF ratio; 7. Triangular index; 8. TINN (ms)	1. 29.13 ± 9.37 vs. 36.17 ± 9.59 (p = 0.0282)<br>2. 24.45 (IQR 14.40–28.55) vs. 27.40 (IQR 23.40–33.15) (p = 0.0452)<br>3. 3.41 (IQR 0.25–7.36) vs. 6.73 (IQR 3.74–12.50) (p = 0.055)<br>4. 66.61 ± 15.65 vs. 65.49 ± 15.30 (p = 0.825)<br>5. 33.33 ± 15.65 vs. 34.49 ± 15.30 (p = 0.8198)<br>6. 1.83 (IQR 1.11–3.88) vs. 1.99 (IQR 1.47–3.39) (p = 0.9883)<br>7. 7.91 ± 2.34 vs. 9.02 ± 1.91 (p = 0.1218)<br>8. 135 ± 40.77 vs. 135 ± 40.77 (p = 0.0404)
Kurtoğlu 2022	cross-sectional study	G1: Patients with a confirmed history of COVID-19 (at 20.0 ± 11.4 weeks) (n = 50)<br>G2: Healthy controls without a history of COVID-19 and vaccination (n = 50)	G1: 40.82 ± 10.31<br>G2: 38.24 ± 12.02	3 months (12 weeks) or more	24-h ECG monitoring (iH-12Plus Holter System)	1. SDNN (ms); 2. SDANN (ms); 3. RMSSD (ms); 4. SDNN Index (ms); 5. pNN50 (%); 6. total power (ms^2); 7. VLF (ms^2); 8. LF (ms^2); 9. HF (ms^2); 10. LF (nu); 11. HF (nu); 12. Triangular index (HRVI)	1. 122.40 ± 30.90 vs. 161.30 ± 30.80 (p < 0.0001)<br>2. 113.90 ± 30.20 vs. 144.70 ± 33.60 (p < 0.0001)<br>3. 1.45 ± 0.16 vs. 1.62 ± 0.18 (p < 0.0001)<br>4. 50.10 ± 13.40 vs. 63.60 ± 14.80 (p < 0.0001)<br>5. 1.03 ± 0.29 vs. 1.23 ± 0.25 (p < 0.0001)<br>6. 3.36 ± 0.24 vs. 3.55 ± 0.21 (p < 0.0001)<br>7. 3.17 ± 0.23 vs. 3.33 ± 0.20 (p = 0.001)<br>8. 2.71 ± 0.31 vs. 2.95 ± 0.28 (p < 0.0001)<br>9. 2.29 ± 0.33 vs. 2.62 ± 0.34 (p < 0.0001)<br>10. 69.60 ± 11.60 vs. 67.80 ± 13.90 (p = 0.482)<br>11. 28.10 ± 11.10 vs. 34.30 ± 16.60 (p = 0.033)<br>12. 17.20 ± 5.05 vs. 20.40 ± 4.95 (p = 0.030)
Liu 2021	cross-sectional study	G1: Discharged COVID-19 patients (n = 186 → 164 analyzed)<br>G2: Matched healthy controls (n = 186 → 166 analyzed)	Not reported	unclear	more than 10-h recording (ballistocardiography-based internet-of-medical-things system)	1. SDNN (ms); 2. SDANN (ms); 3. LF (ms^2); 4. HF (ms^2)	Law data was not reported.<br>long COVID vs. controls (p < 0.05)<br>1. SDNN (ms): <<br>2. SDANN (ms): <<br>3. LF (ms2): <<br>4. HF (ms2): <
Marques 2022	cross-sectional study	G1: Long COVID clinical group (n = 155 → 81 analyzed)	G1: 43.88 ± 10.03<br>G2: 40.69 ± 6.35	3 months (12 weeks) or more	5-min HRV (Polar RS800CX)	1. SDNN (ms); 2. RMSSD (ms); 3. LF (nu); 4. HF (nu); 5.	1. 46.83 ± 133.77 vs. 46.50 ± 29.20 (p < 0.0001)<br>2. 38.25 ± 35.68 vs. 54.90 ± 40.64 (p = 0.000)<br>3. 47.29 ± 18.33 vs. 44.65 ± 20.71 (p = 0.377)<br>4. 52.60 ± 18.33 vs. 55.28 ± 20.69 (p = 0.370)

		G2: Uninfected controls (n = 94)				LF/HF ratio; 6. SD1 (ms); 7. SD2 (ms)	5. 4.22 ± 25.06 vs. 1.26 ± 1.42 (p = 0.235)<br>6. 27.09 ± 25.27 vs. 39.89 ± 28.39 (p = 0.000)<br>7. 35.93 ± 23.32 vs. 51.52 ± 31.79 (p < 0.0001)
Mekhael 2022; Dagher 2022	cross-sectional study	G1: Previous COVID-19 infection (at 171 ± 114 days) (n = 122)<br>G2: Controls who were not diagnosed with COVID-19 (n = 588)	G1: 41.32 ± 15.7<br>G2: 45.99 ± 14.0	171 ± 114 days	5-min HRV (PPG-based smartband)	1. Mean HRV day/person (ms)	1. 38.9 ± 614.4 vs. 44.0 ± 619.2 (p = 0.01)
Shah 2022	cross-sectional study	G1: Previous COVID-19 infection (recovered within 30-45 days) (n = 92)<br>G2: Healthy volunteer controls (n = 120)	G1: 50.6 ± 12.1<br>G2: 51.8 ± 4.2	unclear (recovered within 30-45 days)	1-min HRV (VESTA 301i) responses to orthostatic stress (3-min active standing)	1. RMSSD (ms)	1. 13.9 ± 11.8 vs. 19.9 ± 19.5 (p = 0.01)
Zanoli 2022	cross-sectional study	G1: Previous COVID-19 infection (> 12 weeks) (n = 92)<br>G2: Matched controls (n = 180)	G1: 55 ± 12<br>G2: 55 ± 13	3 months (12 weeks) or more	5-min HRV (Finometer Midi device)	1. LF/HF ratio; 2. Triangular index	1. 1.19 (IQR 0.65–2.13) vs. 1.22 (IQR 0.72–2.09) (p > 0.05)<br>2. 6.74 (IQR 4.92–8.83) vs. 6.92 (IQR 5.33–8.83) (p > 0.05)

Abbreviations. COVID-19, Coronavirus disease 2019; ECG, electrocardiogram; G, group; HF, high frequency band; HRV, heart rate variability; IST, inappropriate sinus tachycardia; IQR, interquartile range; LF, low frequency band; pNN50, proportion of the number of pairs of successive normal-to-normal RR intervals that differ by more than 50 milliseconds divided by the total number of normal-to-normal RR intervals; RMSSD, root mean square of the successive differences; SD, standard deviation of the interbeat interval; SDANN, standard deviation of the averages of normal-to-normal RR intervals; SDNN, standard deviation of normal-to-normal RR intervals; SDNN index, mean of the standard deviations of all normal-to-normal RR intervals for all 5 min segments of the entire recording; TINN, triangular interpolation of normal-to-normal RR intervals; VLF, very low frequency band.
